# Towards Improved Prognostic Scores Predicting Survival in Patients with Brain Metastases: A Pilot Study of Serum Lactate Dehydrogenase Levels

**DOI:** 10.1100/2012/609323

**Published:** 2012-04-19

**Authors:** Carsten Nieder, Kirsten Marienhagen, Astrid Dalhaug, Jan Norum

**Affiliations:** ^1^Department of Oncology and Palliative Medicine, Nordland Hospital, 8092 Bodø, Norway; ^2^Institute of Clinical Medicine, Faculty of Health Sciences, University of Tromsø, 9037 Tromsø, Norway; ^3^Radiation Oncology Unit, Nordlandssykehuset HF, P.O. Box 1480, 8092 Bodø, Norway; ^4^Department of Oncology, University Hospital of Northern Norway, 9038 Tromsø, Norway; ^5^Northern Norway Regional Health Authority, 8038 Bodø, Norway

## Abstract

Accurate prognostic information is desirable when counselling patients with brain metastases regarding their therapeutic options and life expectancy. Based on previous studies, we selected serum lactate dehydrogenase (LDH) as a promising factor on which we perform a pilot study investigating methodological aspects of biomarker studies in patients with brain metastases, before embarking on large-scale studies that will look at a larger number of candidate markers in an expanded patient cohort. For this retrospective analysis, 100 patients with available information on LDH treated with palliative whole-brain radiotherapy were selected. A comprehensive evaluation of different LDH-based variables was performed in uni- and multivariate tests. Probably, the most intriguing finding was that LDH kinetics might be more important, or at least complement, information obtained from a single measurement immediately before radiotherapy. LDH and performance status outperformed several other variables that are part of prognostic models such as recursive partitioning analyses classes and graded prognostic assessment score. LDH kinetics might reflect disease behaviour in extracranial metastatic and primary sites without need for comprehensive imaging studies and is a quite inexpensive diagnostic test. Based on these encouraging results, confirmatory studies in a larger cohort of patients are warranted.

## 1. Introduction

During the last two decades, various research groups have tried to improve our ability to predict overall survival of patients with brain metastases from solid tumours. They have identified a series of independent prognostic factors for survival and, based on these, developed prognostic scores [1–6]. Especially the scores developed on the basis of studies performed by the Radiation Therapy Oncology Group (RTOG) have gained widespread acceptance and were validated by several groups, as recently summarised [7]. These scores named recursive partitioning analysis (RPA) classes [2] and graded prognostic assessment (GPA) [1] both include Karnofsky performance status (KPS), age, and presence of extracranial metastases. Moreover, primary tumour control is included in the RPA classes and number of brain metastases in the GPA score. Despite their clinical usefulness, these scores are not perfect in predicting survival. As demonstrated in a recent analysis [8], even if one combines information from several scores, some patients with predicted short survival might do much better than anticipated while other patients with predicted favourable prognosis might die shortly after treatment.

While factors such as age are straight forward and easy to assign, others are much more complicated and disputable. For example, the term “presence of extracranial metastases” covers a broad spectrum ranging from just one or two small, asymptomatic lung nodules to massive involvement of the liver, possibly with additional lesions in the adrenal glands, bones, and so forth. By just assigning “metastases present” or “absent” potentially valuable information on total tumour load, organ function, and clinical significance is lost. Extensive imaging and restaging on the other hand might not always be indicated, for example, because no change in immediate patient management is expected and/or resources are limited. Therefore, surrogate markers of tumour load, for example, serum biomarkers are an attractive area of research. Our group has recently shown that serum lactate dehydrogenase (LDH) is an important predictor of survival in patients with brain metastases from malignant melanoma [9]. LDH has also been included in a previous analysis that confirmed its independent prognostic impact [10]. Moreover, it influences the malignant melanoma staging system (M1a and M1b require normal LDH). In patients with brain metastases from lung cancer, the prognostic impact of LDH has also been acknowledged [11, 12]. Furthermore, LDH contributes to prognostic models in malignancies such as renal cell cancer, germ cell tumours, and non-Hodgkin's lymphomas. We have therefore decided to select LDH as a promising factor on which we perform a pilot study investigating methodological aspects of biomarker studies in patients with brain metastases, before embarking on large-scale studies that will look at a larger number of candidate markers in an expanded patient cohort.

## 2. Material and Methods

We analyzed patients from a previously described brain metastases database, which is maintained and updated at the first author's institution [8, 9]. For this retrospective pilot study, 100 patients with available information on LDH treated with palliative whole-brain radiotherapy (WBRT; total dose 30 Gy in 10 fractions; no surgery or radiosurgery) during the last 5 years were selected. A backward inclusion was used starting with all patients treated in 2011. Patients were entered on a year-by-year basis until the target group size of 100 was reached. All patients were treated at two different institutions in northern Norway. LDH was part of routine blood chemistry and imaging assessment in patients with newly detected brain metastases treated in these institutions. LDH measurement no older than 2 weeks before the first fraction of WBRT was required. Elevated LDH was defined as ≥205 U/l according to the hospitals' reference value. The prognostic impact of LDH was tested in various fashions (comparison of normal versus elevated LDH; normal versus 1.5x upper limit of normal (ULN) as cut-off; by quartiles and by kinetics) in univariate analyses (log-rank test). Actuarial survival was calculated with the Kaplan-Meier method and compared between different groups with the log-rank test. For multivariate analysis of survival Cox regression analysis was used. A *P* value ≤ 0.05 was considered statistically significant. Sixteen patients were alive at last follow-up (June 01, 2011) with a median follow-up of 8 months (range 1–42). The patient characteristics are shown in Table 1.

## 3. Results

### 3.1. Univariate Analyses

**Figure 1:**
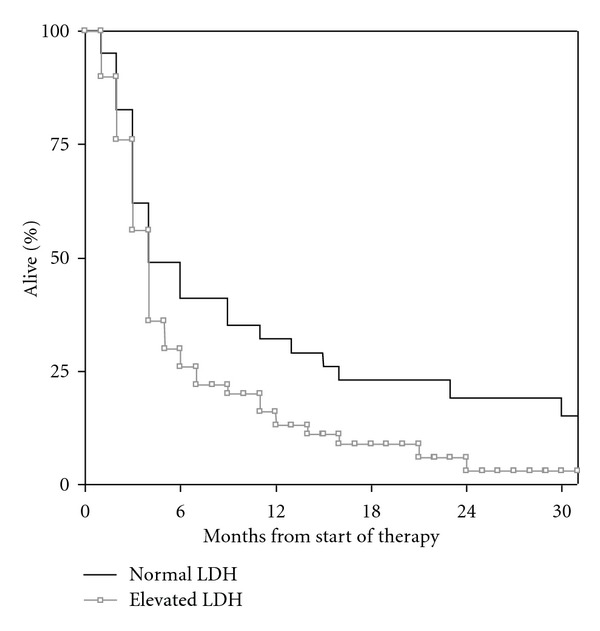
Kaplan-Meier curves for overall survival: normal LDH (*n* = 41) versus elevated LDH (*n* = 59); *P* = 0.037.

**Figure 2:**
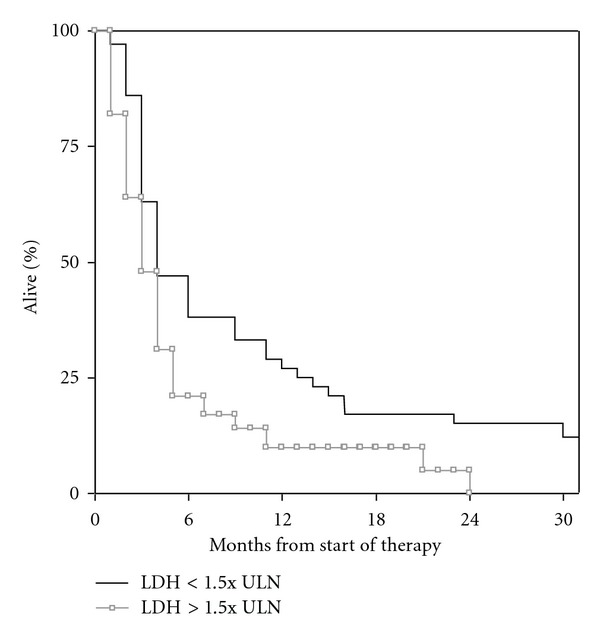
Kaplan-Meier curves for overall survival: LDH less than 1.5x upper limit of normal (*n* = 65) versus LDH higher than 1.5x upper limit of normal (*n* = 35); *P* = 0.013.

**Figure 3:**
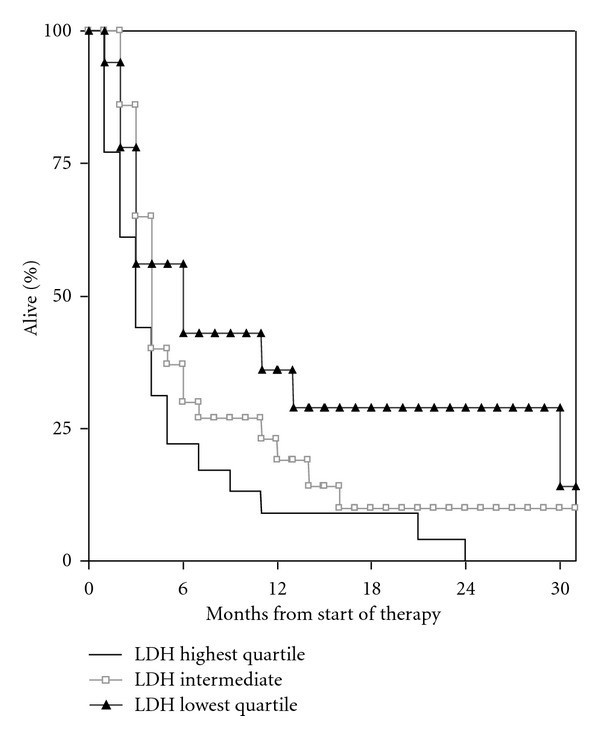
Kaplan-Meier curves for overall survival: LDH highest quartile (**n** = 25) versus LDH lowest quartile (**n** = 25) versus intermediate (**n** = 50); **P** = 0.017.

Patients with normal LDH (<205 U/l) had significantly longer survival (median 4.0 months) as compared to those with elevated LDH (median 3.1 months), *P* = 0.037 (Figure 1). With a different cut-off (1.5x ULN, i.e., LDH <307 U/l), a *P* value of 0.013 was found (median survival 3.0 versus 4.0 months), (Figure 2). When evaluated by quartiles, median survival was 5.5 months in patients with the lowest LDH, 3.4 months in those with intermediate LDH, and 2.8 months in those with the highest LDH, *P* = 0.017 (Figure 3). Given that the lowest *P* value was found when discriminating between patients with LDH higher versus lower 1.5x ULN, this cut-off was chosen for confirmatory analyses stratified by primary tumour type. Consistent trends were seen for all primary tumour types despite low numbers of patients. Regarding lung cancer, median survival was 3.1 months in patients with elevated LDH and 5.6 months in others; *P* = 0.145. Regarding breast cancer, median survival of 3.9 versus 9.0 months was found; *P* = 0.39. Regarding gastrointestinal primary tumours, median survival was 0.9 versus 3.3 months; *P* = 0.215. For melanoma patients, these figures were 1.1 versus 5.5 months; *P* = 0.02.

**Figure 4:**
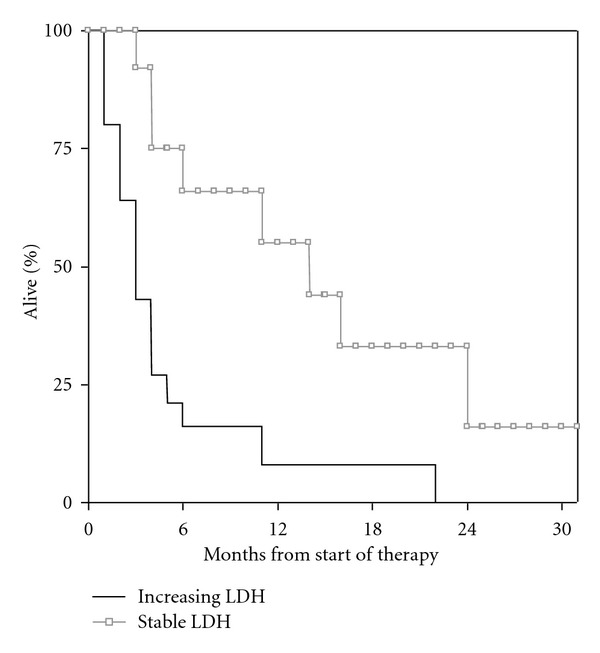
Kaplan-Meier curves for overall survival: LDH increase ≥10% within 2 months (*n* = 20) versus no such increase (*n* = 13); *P* = 0.001.

Data on LDH kinetics were available in 33 patients. We compared the LDH level 2 months before WBRT to that immediately before WBRT. In 20 cases, an increase of at least 10% during the 2 months time period was found. In the remaining 13 cases LDH had been stable, slightly decreasing or increasing <10%. Patients with LDH increase ≥10% had significantly shorter survival (median 2.3 months) as compared to the remaining patients (median 13.5 months); *P* = 0.001 (Figure 4).

### 3.2. Multivariate Analyses

All Cox regression analyses included KPS (continuous variable), age (continuous variable), number of brain metastases detected on magnetic resonance imaging scans (continuous variable), and presence versus absence of extracranial metastases, that is, all parameters that determine the GPA classification. When adding LDH (continuous variable) to these 4 parameters, only KPS (*P* = 0.0001) and LDH (*P* = 0.002) retained statistical significance. Comparable results were seen when LDH was entered as categorical variable with cut-off 1.5x ULN. The *P* values were 0.0001 for KPS and 0.001 for LDH, respectively. When both LDH variables were included, only the one with cut-off 1.5x ULN retained significance (KPS *P* value 0.0001, LDH by cut-off 1.5x ULN *P* value 0.019, LDH as continuous variable *P* = 0.188).

The model based on LDH kinetics included only 33 patients, as mentioned previously. LDH was entered as categorical variable with cut-off ≥10% increase during a time period of 2 months. In addition, LDH by cut-off 1.5x ULN was included. Again, KPS was the most important prognostic factor, *P* = 0.001, followed by LDH kinetics, *P* = 0.002, and LDH by cut-off 1.5x ULN, *P* = 0.01. As in the previous analyses, age, number of brain metastases, and extracranial metastases were not significant.

In order to exclude any confounding influence of primary tumour control, which is included in the RPA scoring system, these analyses were repeated with KPS (continuous variable), age (continuous variable), number of brain metastases (continuous variable), presence versus absence of extracranial metastases, and controlled versus uncontrolled primary tumour. Again, KPS and LDH outperformed all other variables and were the only ones to obtain statistical significance (details not shown).

## 4. Discussion

This pilot study, which was performed in a homogenously treated patient population, is to the best of our knowledge the first one that included a comprehensive evaluation of different LDH-based variables. It represents a first step towards analyses of different surrogate markers in larger patient groups and confirms that such studies are warranted because biomarkers obviously might add important prognostic information. Moreover, the present data provide important insights, which will influence the design of our future studies. Probably, the most intriguing finding was that LDH kinetics might be more important, or at least complement, information obtained from a single measurement immediately before WBRT. Since the number of patients with available data on LDH kinetics was limited, confirmatory studies are needed. A possible explanation for the impact of LDH kinetics is that this parameter reflects disease progression in extracranial sites, and that patients with well controlled or even absent extracranial disease have longer survival. In general, LDH is upregulated in many tumours and lactate enhances the degree of tumour malignancy [13]. LDH level might also increase with tumour volume. Compared to imaging studies, LDH is an inexpensive diagnostic test. Lagerwaard et al. [3] have analysed a much larger patient cohort with brain metastases from different primary tumours (*n* = 1292). Apparently, their study is the largest one that included LDH as a potential prognostic factor. Yet the only comparison was made between patients with normal versus elevated LDH (median survival 4 versus 2.2 months). Multivariate analysis confirmed that LDH influenced survival (risk ratio 1.55, 95% confidence interval 1.32–1.81, *P* < 0.001). A very similar result was obtained in multivariate analysis of 692 patients with brain metastases from malignant melanoma [14]. Again normal versus elevated LDH was included. The hazard ratio was 1.6 (95% confidence interval 1.3–2.0, *P* = 0.00013). In terminally ill cancer patients referred for palliative radiotherapy (with or without brain metastases, different primary tumour types), LDH was also associated with survival outcome in multivariate analysis [15].

Our study was not powered to confirm the independent prognostic significance of LDH in subgroups with different primary tumours. However, exploratory subgroup analyses were all in agreement with the main analysis, which included 100 patients. While previous studies already suggested that LDH predicts survival in patients with brain metastases from malignant melanoma and lung cancer [9–12, 14], our data suggest that LDH could also be useful in patients with breast and gastrointestinal primaries. Future studies should address whether LDH could replace other factors that are included in the GPA score, for example, extracranial metastases, and whether this would impact on the accuracy of survival predictions.

Avoiding overtreatment in patients with poor prognosis is crucial when trying to avoid unnecessary complications and achieve maximum value for health care budget. The challenge is to assign the right patient to the right treatment, with clear objectives being set upfront, for example, palliation of symptoms in the terminal phase of disease. Robust and reproducible prognostic models might guide clinical decision making.

## Figures and Tables

**Table 1:** Pretreatment characteristics of all 100 patients included in this study.

Parameter	Number and %
Elevated serum lactate dehydrogenase (LDH)	59
Normal LDH	41
Extracranial metastases absent	14
Extracranial metastases present	86
Controlled primary tumour	71
Uncontrolled primary tumour	29
Solitary brain metastasis	17
Two or three brain metastases	44
More than 3 brain metastases	39
Female sex	70
Male sex	30
Lung cancer	36
Breast cancer	34
Gastrointestinal cancer	11
Malignant melanoma	10
Kidney cancer	4
Other primary cancer	5
Median Karnofsky performance status	70% (range 30–100)
Median age	62 years (24–85)
Median number of brain metastases	3 (1–50)
Median LDH	228 (99–3190)
